# At-Home Foscarnet Administration in Patients with Cytomegalovirus Infection Post-Allogeneic Stem Cell Transplantation: A Unicentric, Safe, and Feasible Program

**DOI:** 10.3390/ph16121741

**Published:** 2023-12-18

**Authors:** Sonia Ruiz-Boy, Alexandra Pedraza, Marta Prat, Maria Queralt Salas, Esther Carcelero, Gisela Riu-Viladoms, María Suárez-Lledó, Inés Monge-Escartín, Luis Gerardo Rodríguez-Lobato, Alexandra Martínez-Roca, Montserrat Rovira, Carmen Martínez, Cristina Gallego, Álvaro Urbano-Ispizua, Joan Sánchez, María Ángeles Marcos, Francesc Fernández-Avilés

**Affiliations:** 1 Pharmacy Service, Division of Medicines, Hospital Clínic de Barcelona, 08036 Barcelona, Spain; maprat@clinic.cat (M.P.); ecarcele@clinic.cat (E.C.); griu@clinic.cat (G.R.-V.); monge@clinic.cat (I.M.-E.); 2 Hematopoietic Stem Cell Transplantation Unit, Hematology Department, Institute of Cancer and Blood Diseases, Hospital Clínic de Barcelona, IDIBAPS (Institut De Investigacions Biomèdiques August Pi i Sunyer), Josep Carreras Institute, 08036 Barcelona, Spain; acpedraza@clinic.cat (A.P.); mqsalas@clinic.cat (M.Q.S.); msuarezl@clinic.cat (M.S.-L.); lgrodriguez@clinic.cat (L.G.R.-L.); apmartinez@clinic.cat (A.M.-R.); mrovira@clinic.cat (M.R.); cmarti@clinic.cat (C.M.); aurbano@clinic.cat (Á.U.-I.); ffernand@clinic.cat (F.F.-A.); 3 Blood Bank Department, Biomedical Diagnostic Center, Blood and Tissue Bank, Hospital Clínic de Barcelona, 08036 Barcelona, Spain; 4 Home Care and Bone Marrow Transplantation Unit, Department of Hematology, Hospital Clínic de Barcelona, 08036 Barcelona, Spain; cgallego@clinic.cat; 5 Financial-Economic Department, Institute of Cancer and Blood Diseases Hospital Clínic de Barcelona, 08036 Barcelona, Spain; jsperez@clinic.cat; 6 Microbiology Department, Hospital Clínic de Barcelona, University of Barcelona, ISGlobal, CIBERINFEC (Centro de Investigación Biomédica En Red enfermedades INFECciosas), 08036 Barcelona, Spain; mmarcos@clinic.cat

**Keywords:** Cytomegalovirus, allogeneic stem cell transplantation, foscarnet, at-home model

## Abstract

Cytomegalovirus (CMV) infection is a relevant cause of morbimortality in patients receiving allogeneic stem cell transplantation (allo-HCT). Foscarnet (FCN) is an effective drug against CMV administered intravenously and usually on an inpatient basis. The Home Care Unit (HCU) for hematologic patients at our hospital designed an at-home FCN administration model to avoid the hospitalization of patients requiring FCN treatment. This study analyzes whether the at-home administration of FCN is as safe and effective as its hospital administration. We collected and compared demographic, clinical, analytical, and economic data of patients with CMV infection post-allo-HCT who received FCN in the hospital (*n* = 16, 17 episodes) vs. at-home (*n* = 67, 88 episodes). The proportions of patients with cured CMV infections were comparable between the two groups (65.9% vs. 76.5%, *p* = 0.395). The median duration of FCN treatment was 15 (interquartile range [IQR] 9–23) and 14 (IQR 11–19) days in the HCU and inpatient cohorts, respectively (*p* = 0.692). There were no significant differences in the FCN toxicities between groups except for hypocalcemia (26.1% vs. 58.8%, *p* = 0.007), which was more prevalent in the inpatient cohort. A significant cost-effectiveness was found in the HCU cohort, with a median savings per episode of EUR 5270. It may be concluded that home administration of FCN is a safe, effective, and cost-efficient therapeutic option for patients with CMV infection and disease.

## 1. Introduction

Cytomegalovirus (CMV) infection is one of the major causes of morbimortality in allogenic hematopoietic cell transplant (allo-HCT) recipients [1]. The current incidence of CMV disease and infection in patients receiving allo-HCT is 2–10% and 35–76%, respectively [1,2]. The reported spectrum of CMV infection ranges from CMV reactivation without organ involvement, through CMV end-organ diseases (such as gastroenteritis, retinitis, esophagitis, pneumonia, or encephalitis) and CMV syndrome, to disseminated CMV disease and death. In addition, CMV reactivation can indirectly affect graft failure or immunosuppression, which may result in concurrent bacterial and/or fungal infections [3,4,5].

One of the most commonly used drugs to treat CMV infection or disease is foscarnet (FCN). This is a second-line drug used primarily when ganciclovir/valganciclovir treatment is contraindicated, ineffective, or has caused significant toxicity to the patient. FCN is a pyrophosphate analog that inhibits the DNA polymerases of all herpes viruses, with an effective action against CMV [6]. FCN administration is performed through an intravenous infusion in the hospital [7]. The efficacy of FCN for controlling CMV infection and disease has been demonstrated in different publications [1,2]. However, its use has also been associated with relevant toxicities such as kidney injury and dyselectrolytemia, genital toxicity, and gastrointestinal discomfort [6,8]. All these drug-related side effects result in the significantly difficult administration of FCN on an outpatient basis. 

Since 2000, a complex and multidisciplinary Home Care Unit (HCU) has been designed and implemented at our institution for the administration of outpatient intravenous treatments, the management of chemotherapy-induced myelotoxicity, and the conduction of outpatient autologous and allo-HCT [9,10,11]. In this way, it is possible to administer highly complex drugs to patients in the comfort of their homes, as well as to control their vital signs, clinical signs, and analytical monitoring [10]. In 2011, the HCU team designed an at-home FCN administration model to minimize hospital admission, which implies the use of this antiviral in patients with CMV infection. A systematic review concluded that outpatient HCT programs are safe and effective, and their main advantages include significant cost reduction, alleviating constraints on chronic bed shortages, and facilitating patient convenience [12]. However, no articles have yet focused specifically on the home administration of FCN for CMV treatment. This study aims to evaluate the safety and effectiveness of home administration of FCN. For this purpose, the results obtained in the home program are compared with those obtained in a control cohort of patients receiving FCN in the hospital. 

## 2. Results

### 2.1. Patient and Transplant Characteristics

The main demographic and transplant characteristics are described in Table 1. A total of 105 episodes, corresponding to 82 adult recipients of allo-HCT with CMV infection/disease were included in this study. Patients received intravenous FCN treatment at-home (*n* = 67, 88 episodes) or at the hospital (*n* = 16, 17 episodes). One patient (1.2%) presented episodes in both cohorts. 

Of the 82 patients included, 53.7% were males and 56.1% were older than 50 years. Most patients had a body surface area within normality, few extra comorbidities, and high functional capacities. Acute leukemia/myelodysplastic syndrome was the most prevalent baseline diagnosis (63.4%). A total of 33.0% of adults received myeloablative conditioning regimens, 15.9% received grafts from identical family donors, 67.0% from unrelated donors, and 15.9% from haploidentical donors. 

The clinical and transplant characteristics of both cohorts were well balanced, except for Graft-Versus-Host-Disease (GVHD) prophylaxis, donor type, and CMV serological risk. In the HCU cohort, unrelated donors prevailed. In the inpatient cohort, unrelated donors and siblings were equally represented. GVHD prophylaxis included mostly methotrexate and antithymocyte globulin in the inpatient cohort, whereas posttransplant cyclophosphamide predominated in the HCU cohort. In the inpatient cohort, a higher percentage of patients with a high CMV risk was observed than in the HCU cohort.

### 2.2. Antiviral Treatment Characteristics and Patient Outcomes 

As reported in Table 2, most patients in both cohorts had only one episode of FCN administration (81.7%). CMV disease was present in 11.4% of episodes, with gastrointestinal involvement being the most frequent (50.0%). Neutrophil count < 2.0 × 10^9^/L at the start of episodes was found in 54.5% (*n* = 48) and 41.2% (*n* = 7) of the episodes in the HCU and inpatient cohorts, respectively. The glomerular filtration rate at the start of FCN was below 60 mL/min in twenty-four episodes (27.3%) of the HCU cohort and five episodes (29.4%) of the inpatient cohort. Creatinine clearance was ≤1.4 mL/min/kg in 56 (63.6%) and 12 (70.6%), respectively. 

The most common treatment schedule was 60 mg/kg/12 h of FCN in both cohorts (65.9% in HCU and 64.7% in inpatient).

There were 15 episodes (17.0%) in the HCU cohort in which the patient started FCN treatment in the hospital before discharge to the at-home program. In nine cases (10.2%), CMV reactivation occurred during the transplant procedure. The median number of days of FCN treatments in the hospital for these patients was 5 days. 

Many patients in the HCU cohort (61.4%) were started directly on FCN, mainly because they had neutropenia, thrombocytopenia, or pancytopenia. In contrast, in the inpatient cohort, treatment had been initiated mostly with valganciclovir (76.5%) and later switched to FCN due to myelotoxicity or failure of valganciclovir therapy. In no case was it necessary to reduce/discontinue immunosuppressive therapy when starting treatment with FCN. The dose of immunosuppressive drugs was adjusted according to pharmacokinetic monitoring.

In 22 of the HCU cohort episodes (25.0%), the patient had to go either to the emergency department (ER) or be admitted to the hospital. In two cases, this happened twice. Of the twenty-four cases (twenty admissions and four ER visits), the main reasons were: complications of a non-CMV infection (45.8%), related to FCN administration (16.7%), severe GVHD (12.5%), and disease progression (8.3%). Complications related to FCN administration were catheter bacteremia (two cases), technical failure of the FCN administration pump (one case), and persistence of viral load in addition to worsening renal function (patient admitted for combined FCN and ganciclovir treatment and renal function monitoring). Of the twenty hospitalizations, in two cases FCN was discontinued at the time of admission (limitation of therapeutic effort and CMV infection resolution), in twelve cases the patient finished FCN during admission, and in six cases the patient was discharged and returned to the HCU (median number of days on admission in the latter cases: four; range 2–28). Two of the patients who were admitted required intubation and an ICU stay (in both cases due to a severe respiratory infection ± septic shock), and in one of the cases, the patient died of the same respiratory infection and other complications.

The main reasons why patients in the inpatient cohort were not treated at-home were an at-home program not yet available (70.6%) and the patient’s residence outside the scope of the HCU (23.5%). Of the patients in the inpatient cohort, two were admitted to the ICU due to a severe respiratory infection and associated septic shock. Both patients died due to these complications.

The other reasons to end treatment with FCN in the HCU cohort were dyselectrolytemia that was difficult to control in a patient who already had it before starting FCN (*n* = 1), loss of venous access (*n* = 1), change to cidofovir because of hemorrhagic cystitis due to BK virus (*n* = 1), and disease progression (*n* = 2).

No significant differences were found with respect to treatment duration, CMV viral load, infections developed during FCN treatment, reason for FCN termination, and types of toxicities causing discontinuation. The most frequent type of infection occurring during treatment with FCN in both cohorts was BK virus cystitis, followed by febrile syndrome without focus.

### 2.3. Toxicity Outcomes

Toxicity data are given in Table 3. No significant differences were found in any of the toxicities studied, except for episodes of hypocalcemia, which were more prevalent in the inpatient cohort (26.1% vs. 58.8%, *p* = 0.008).

Renal toxicity: of the sixteen de novo AKIs and the five CKD exacerbations in the HCU cohort and the two de novo AKIs and one CKD exacerbation in the inpatient cohort (corresponding to twenty (29.9%) and three patients (18.8%), respectively), 13 were in grade I and 11 were in grade II. In addition, there were two cases (one in each cohort) where the patients developed proteinuria (grade I in the HCU cohort and grade II in the inpatient cohort) without associated AKI, which was the cause of the discontinuation of FCN treatment in both cases. In all cases but one, the patient was receiving at least one other nephrotoxic drug (mostly tacrolimus, fluconazole, or voriconazole). During the same FCN treatment, there were four grade I de novo AKIs that resolved, one grade II de novo AKI that progressed to a grade I (all HCU cohort cases), and two CKD exacerbations that also resolved (one per cohort). The median time of onset between starting treatment with FCN and the worst creatinine value was 14 (interquartile range [IQR]: 8–22) days.

Genital ulcers: in the HCU cohort, eighteen patients developed genital ulcers (26.9%). Of these, two were presumably not related to FCN (in one case, the patient also had genital herpes, and after stopping FCN, it worsened, and in the other, the patient had also had an ulcer for about a month already caused by *Klebsiella* and *Pseudomonas*). In nine of the eighteen ulcers (50.0%) and in five of the eleven non-ulcer dysurias (45.5%), the patient also had BK virus cystitis, in four and two cases of hemorrhagic type, respectively. In the inpatient cohort, one of the two patients who developed ulcers also had BK virus hemorrhagic cystitis (50.0%). There was a significant positive association between having cystitis (hemorrhagic or not) due to the BK virus and the development of dysuria or ulcers (*p* < 0.001). The median time from initiation of FCN treatment to onset of urethral symptoms was 7 (IQR: 5–13) days. From the moment the patient reported dysuria, the institutional protocol was followed.

Gastrointestinal toxicity: of the thirty episodes in the HCU cohort and the five in the inpatient cohort [corresponding to twenty-six (38.8%) and five (31.2%) patients, respectively] with digestive adverse effects, thirty-three were grade II and the remaining two were grade I. The main symptomatology was nausea, usually accompanied by vomiting, present in 31 of the episodes (88.6%). In 18 of the episodes (51.4%), this symptomatology resulted in an intake of less than 50% of usual. Medication (antiemetics, especially) was required in 31 cases (88.6%), in which 10 symptoms did not improve despite the prescribed drug. There were three other patients in the HCU cohort with gastric symptoms presumably unrelated to FCN (CMV esophagitis, pseudo-occlusive symptoms, and adverse effects of an oral antibiotic). The median time from initiation of FCN treatment to onset of gastric symptoms was 3 (IQR: 1–5) days.

Infusion reactions: there were thirteen episodes in the HCU cohort and four in the inpatient cohort [corresponding to 10 (14.9%) and four (25.0%) patients, respectively] suffering from infusion reactions, all of which were grade I. The main symptomatology was paresthesia (82.3%), and the median number of days from FCN initiation to the onset of infusion reactions was 2 (IQR: 1–5) days.

Dyselectrolytemia: there were ten episodes of de novo hyponatremia in the HCU cohort and four in the inpatient cohort [corresponding to ten (14.0%) and four (25.0%) patients, respectively and being two cases of grade II]; 49 episodes of de novo hypokalemia in the HCU cohort and 11 in the inpatient cohort (corresponding to 41 (61.2%) and 10 (62.5%) patients, respectively, and being twenty-four of grade II and one of grade III); thirty-three episodes of de novo hypomagnesemia in the HCU cohort and four in the inpatient cohort (corresponding to twenty-six (38.8%) and four (25.0%) patients, respectively and being one of grade II and one of grade III); and twenty-one episodes of de novo hypocalcemia (albumin-adjusted) in the HCU cohort and ten in the inpatient cohort (corresponding to twenty (29.9%) and nine (56.3%) patients, respectively, and being eleven of grade II). In addition, there were two episodes of worsening hypokalemia from grade I to II (corresponding to two patients in the HCU cohort, 3.0%), five episodes of worsening hypomagnesemia from grade I to II (corresponding to four patients (6.0%) in the HCU cohort and one (6.3%) in the inpatient cohort), and two episodes of worsening hypocalcemia from grade I to II (corresponding to two patients in the HCU cohort, 3.0%). A total of 78.6% of hyponatremia, 61.3% of hypokalemia, 50.0% of hypomagnesemia, and 81.8% of hypocalcemia could be resolved during the FCN treatment period through intensive intravenous electrolyte(s) replacement.

Other toxicities: There was no FCN-related cardiac toxicity in either cohort. One patient in the HCU cohort experienced a grade I increase in GGT during treatment with FCN, and another subject reported grade I insomnia that he related to FCN.

### 2.4. Pharmacoeconomic Analysis

Economic data are given in Table 4. We found that treating patients in the HCU cohort was significantly less expensive than in the inpatient cohort. Significant economic savings were found, including the admission costs of those patients in the HCU cohort who started FCN during hospitalization before discharge or those who had to be admitted for complications during FCN treatment at HCU.

In the HCU cohort, the most important costs were staff costs, followed by pharmacy costs; while in the hospital, the most significant costs were the costs of the stay in the health center, followed by the costs of either pharmacy or diagnostic tests.

In the inpatient cohort, four patients with significant complications were excluded, whose price was significantly higher than the others: the two patients who were admitted to the ICU, one patient with CMV myelopathy, and one with organized pneumonia who underwent extensive testing to reach a diagnosis.

## 3. Discussion

In this retrospective study, we evaluated the feasibility and reproducibility of home administration of FCN for the treatment of CMV infection or disease in patients who underwent allo-HCT. We aimed to demonstrate that home administration of FCN is as safe and effective as hospital administration while also being more economical. The results of our study show that home administration of FCN is a viable option for the treatment of CMV infection in post-allo-HCT patients.

There is very little literature that has described the use of FCN at-home and no other published article has compared home-based versus hospital-based FCN as a primary endpoint. In addition, this is the first article on the home administration of FCN that includes an economic study. There are two case reports from the late 1990s where FCN was successfully used at-home to treat CMV retinitis in AIDS (acquired immunodeficiency syndrome) patients [13,14]. On the other hand, there is an article comparing continuous vs. intermittent infusion of FCN at-home and in the hospital, with no differences in safety or efficacy depending on the mode of administration [15].

The patient profile found is similar to that of other articles with FCN [15,16]. Patients were predominantly transplanted in 2011–2013 (inpatient cohort) and 2016–2018 (HCU cohort). Most of the inpatient cohort episodes are from before the creation of the HCU. As the recommendations for prophylaxis for GVHD evolve over the years [17], as well as unrelated donor transplantation techniques [18], we can see significant differences in these aspects between the two cohorts. We do not believe that these differences, as well as those found with CMV serological risk, could interfere in any way with efficacy or safety results.

The effectiveness outcomes of the study show the home administration model for FCN is feasible and can effectively manage CMV infection in this patient population, leading to favorable clinical outcomes. Several studies have shown that parenteral administration of antibiotics at-home is as effective as in the hospital [19,20,21]. However, two recent systematic reviews reported that the evidence for the potential advantages of home versus hospital administration of parenteral antibiotics is still low, and more studies are needed to increase it [22,23].

Regarding safety, the observed toxicities were consistent with the known side effects of FCN reported in previous studies [15,24,25]. The close monitoring of renal function, electrolyte levels, and symptoms by the home care team contributed to the early detection and management of toxicities, ensuring patient safety during home administration of FCN. Notably, the incidence of nephrotoxicity, including AKI and exacerbations of CKD, was relatively low in both cohorts. However, even with adequate hydration and close monitoring of renal function, the high nephrotoxicity of foscarnet, together with the concomitant use of other nephrotoxic drugs necessary for the prophylaxis of GVHD or viral/fungal infections, makes it almost impossible to decrease the rate of renal damage to values close to zero. A significant association was found between having BK virus cystitis and developing dysuria or ulcers. BK virus reactivation is common after allo-HCT and has been associated with the presence of hematuria, dysuria, bladder spasm, and increased urinary frequency, among others, causing serious morbidities [26]. Improvements in virus detection techniques, as well as the growing importance of this virus with the latest publications, mean that the incidence of BK virus in post-transplant patients is increasing [27].

The cost savings found in the study highlight the potential economic benefits of home administration of FCN, which can alleviate the financial burden associated with hospitalization. Outpatient models for hematopoietic stem cell transplant recipients, as well as studies of outpatient antibiotic stewardship, also demonstrate substantial savings [12,22,28].

The present study does not include patient satisfaction and quality of life surveys. However, it has been observed in systematic reviews and meta-analyses that patients and/or relatives have a better quality of life and are more satisfied when treated at-home than in the hospital [12,22,28].

The introduction of letermovir will presumably decrease the use of FCN for post-allo-HCT patients. However, this is a high-cost drug that is not accessible to all populations; it has multiple interactions, and it has only been approved for prophylaxis of CMV infection and disease in CMV-seropositive recipients of an allo-HCT [29]. It is important to note that the first-line drugs for prevention, preemptive therapy, and treatment of CMV are ganciclovir and its oral prodrug, valganciclovir. However, the onset of myelotoxicity and, less frequently, the development of ganciclovir-resistance or treatment refractoriness limit the administration of this medication in the allo-HCT setting [30,31]. In these cases, FCN is the drug indicated, and home administration of FCN can be considered an attractive alternative for the treatment of this complication [6,15].

The main result observed in our analysis is that FCN can be administered in an outpatient basis but in the setting of an at-home program with a high degree of expertise capable of early detection of potential adverse events derived from the administration of this drug. Notice, however, that the retrospective design of the present analysis and the fact that all patients were provided by a single center may limit the extension of these conclusions to other institutions. Moreover, the limited number of adults included in the cohort of patients treated on an inpatient basis may have limited the power of the statistical comparisons. Further analyses, including a larger sample size of patients, would be conducted to better address this limitation.

## 4. Materials and Methods

### 4.1. Patient Selection

The present study includes 105 consecutive episodes of CMV infection and disease treated with FCN occurring in 82 patients undergoing allo-HCT at the Hospital Clinic de Barcelona between January 2008 and December 2021. The data was collected retrospectively through chart reviews. This study was approved by the center’s Research Ethics Committee and complies with the basic principles of the Declaration of Helsinki. 

#### 4.1.1. HCU Cohort Criteria

The eligibility criteria for the at-home FCN program by the HCU included: CMV infection or disease with FCN therapy indication, regardless of previous kidney function; Eastern Cooperative Oncology Group (ECOG) performance status of 0 to 2; resided at less than 60 min and less than 30 km to the hospital; a central venous catheter (CVC) of peripheral, subcutaneous, or central insertion with one or two lumens; and understanding of program logistics by the patient and caregiver.

All patients who received all or part of the treatment with FCN in the HCU within the at-home FCN program were included in the HCU cohort.

#### 4.1.2. Inpatient Cohort Criteria

Only patients whose primary cause of admission was the FCN treatment for CMV infection or disease were eligible to be included in this cohort. Patients who were admitted to the hospital for a different cause than CMV reactivation and, during admission, required FCN treatment were excluded. 

### 4.2. Foscarnet Preparation

For at-home administration, the pharmacy department prepared each prescribed dose of FCN in ethylene vinyl acetate vacuum bags. These doses were prepared in sterile conditions under class II biosafety cabinets. The physicochemical stability of these preparations was 7 days at room temperature, so several doses of treatment (for the same patient or different patients) could be prepared in a single day. The program used for the preparation of cytostatic and intravenous mixtures allowed traceability of doses, batches, and expiration dates. During the preparation, a barcode gravimetric control was carried out to minimize preparation errors and improve patient safety.

In the inpatient area, the nurses administered FCN directly from the prefabricated vials which contain 6 g/250 mL of FCN, discarding the amount of medication that is left over after the prescribed dose.

If the patient (in hospital or at-home) did not have a central line, the preparation was always carried out in the pharmacy department. In that case, the prescribed amount of drug was diluted in 250 mL of a 0.9% saline solution.

### 4.3. Foscarnet Administration Model

In the HCU, patients received a 1–2 h FCN infusion at 60 mg/kg/12 h for an estimated glomerular filtration rate (eGFR) > 50 mL/min. The first dose was administered at the hospital in the admitted area or daycare unit. For the following doses, a nurse specializing in hematology care visited patients once or twice per day, according to the number of lumens on the patient’s CVC. All patients received hydration with 1 L of 0.9% saline solution, 1 g calcium gluconate, 20 mEq potassium chloride, and 1.5 g magnesium sulfate, and then the FCN infusion. The home care team performed laboratory tests three times a week to control serum electrolyte levels and kidney function. The viral PCR monitoring was performed weekly once the treatment had started. According to the analytical results, the HCU hematologist modified the dosing schedule of FCN, as well as the hydrating solution and intravenous electrolytes.

An electronic infusion pump (BII) CADD-Legacy^®^ to connect the hydration solution for 8 h was used for patients with a single lumen catheter. In these cases, the nurse made two home visits on the same day. The first is to administer morning parenteral hydration and the FCN dose, as well as to connect a new portable electronic pump with hydration. The second visit is to administer the corresponding dose of FCN and reconnect the hydration solution before the next dose of FCN.

For patients with a two lumen catheter, the nurse made one home visit each day. In one lumen, the morning dose of FCN was connected, and simultaneously, in the other lumen, the hydration solution was connected. When the infusion was finished, a BII was connected at each lumen, one to administer the hydration solution for 8 h previously, the next FCN, and the other to administer the afternoon FCN dose.

At every visit, the nurse team taught patients or caregivers to remove the BIIs after treatment, monitor alarm signs, perform good genital hygiene, and evaluate drug tolerance.

In the case of administration at the FCN hospital, the procedure was the same as described above, except that the patient received all doses of foscarnet in the hospital and did not need to remove the BIIs himself.

### 4.4. Foscarnet Treatment Scheme

The FCN treatment schedules used in both cohorts were in accordance with the internal protocol of the hospital’s hematology service. This protocol is updated at least every 2 years, and its information is in accordance with the recommendations in the drug’s technical data sheet and the American and European guidelines, as well as in the latest reviews published on this subject. Table S3 of the supplementary material contains a summary of the dosing schedules for FCN indicated in this protocol.

### 4.5. Data Collection

All the data collected for the study are listed in Table S1 of the supplementary material. Data were collected from the electronic medical record.

### 4.6. Main Definitions

CMV infection was defined as CMV-PCR above 1000 UI/mL or two consecutive values of PCR rising. CMV disease requires a diagnosis by tissue biopsy or immunostaining (except for CMV retinitis), in addition to CMV DNAemia and symptomatology [4]. The virologic cure was defined as the achievement of two consecutive undetectable CMV PCR results at least 5 days apart. Failure of treatment was defined as the inability to achieve a more than 1 log_10_ decrease in CMV viral load after 14 days or more of anti-CMV treatment.

The serological risk of CMV infection/disease post-allo-HCT is classified according to the pre-transplant serostatus (+ or −) of both the donor (D) and recipient (R). The D−/R+ combination is associated with high risk, D+/R+ with intermediate risk, and D+/R− and D−/R− with low risk [1,3].

Chronic kidney disease (CKD) is defined, according to the latest update of the 2012 Kidney Disease Improving Global Outcomes (KDIGO) guideline, as the presence for at least 3 months of an eGFR lower than 60 mL/min/1.73 m^2^ or kidney injury markers. The degree of severity of CKD has been determined according to the same guidelines [32].

Acute kidney injury (AKI) was defined and classified into three stages according to the KDIGO guideline [33]. Stages are described in supplementary material (Table S2). Within AKI, AKI de novo (those patients who did not have CKD before receiving FCN) has been differentiated from CKD exacerbations (those patients who, before receiving FCN, had CKD and, during treatment, their renal function worsened, thus fulfilling the AKI criteria according to the KDIGO guidelines).

For the classification of the other toxicities collected into the different degrees of severity, the Common Terminology Criteria for Adverse Events (CTCAE) v5.0 was used [34]. Table S4 of the supplementary material contains a definition of the different stages of the toxicities mentioned in the article.

In the supplementary material (Table S5), there are descriptions of the different index and functionality scales used in this study, as well as their bibliographic references.

### 4.7. Statistical Analysis

The primary endpoint of this study was the percentage of patients with a cure for the CMV infection. Secondary endpoints were the percentage of patients with FCN toxicity (in total and organ-specific), the median number of days on FCN treatment, and the median cost per episode with FCN.

To perform the analysis, the study cohort was divided into two groups according to the modality of FCN administration: in-patient vs. out-patient. Categorical variables were presented as counts and percentages, while continuous variables were described using the mean ± standard deviation or the median [interquartile range], depending on the data distribution. A chi-squared test was used for categorical variables. Numerical variables were analyzed using Student’s t-tests or Wilcoxon tests, depending on the variable distribution.

A cost-minimization analysis was carried out. The price of admission to the HCU and/or hospital was recorded, including the cost of medical care, drugs received, analyses or other tests performed, and transfusions. We compared: (a) the total cost of the FCN treatment episode (including extra costs not related to the administration of FCN, such as admission to the ICU due to complications or the cost of allo-HCT); and (b) the total cost and cost per day of only the period where the patient received FCN (excluding in the HCU cohort, the stages where the patient received FCN at hospital if these existed, and in the inpatient cohort, those patients who suffered major complications during admission and that this resulted in a significant increase in costs, which was defined as a more than two-fold increase in costs, compared to the others).

All tests were two-tailed, and *p*-values of <0.05 were considered statistically significant. Statistical tests were performed using R software 4.3.1, while the other calculations were carried out with Microsoft Excel Office 2019.

## 5. Conclusions

Our study demonstrates the feasibility and reproducibility of home administration of FCN for the treatment of CMV infection in post-allo-SCT patients. The home administration model was found to be as safe and effective as hospital administration, with comparable rates of virologic cure and similar toxicity profiles. Similar virological cure rates were obtained, as well as similar treatment durations. Only a low percentage of patients receiving FCN at-home had to be admitted to the hospital. In addition, the frequencies of the most common adverse effects of FCN, as well as their degrees of severity and the percentage of treatment discontinuation due to them, were also comparable between the two cohorts. Finally, substantial economic savings were found in home administration compared to hospital administration. Further studies and prospective trials are warranted to confirm these findings.

## Figures and Tables

**Table 1:** Demographic variables, transplant characteristics, and clinical variables of patients prior to transplantation.

Data, *n* (%)	Total Group (*n* = 82 *)	Home Care Unit Patients (*n* = 67)	Inpatients (*n* = 16)	*p*
Age (years); median, range	55.0, 18–68	56.0, 18–68	50.5, 18–65	0.283
Sex, male	44 (53.7)	37 (55.2)	7 (43.8)	0.416
Body surface area; mean ± SD	1.8 ± 0.2	1.8 ± 0.2	1.7 ± 0.2	0.404
Non-hematological comorbidities, median (IQR)	1 (1–2)	1 (1–2)	1 (1–2)	0.548
HCT-IC ≥ 3	9 (11.0)	8 (12.0)	1 (6.3)	0.514
Karnofsky Performance Status Scale = 60–80	17 (20.7)	14 (20.9)	3 (18.8)	0.853
ECOG Performance Status Scale ≥ 2	10 (12.2)	7 (10.4)	3 (18.8)	0.356
Disease risk index				0.090
Low	20 (24.4)	13 (19.4)	0 (0.0)	
Intermediate	46 (56.1)	37 (55.2)	9 (56.2)	
High/very high	13 (15.8)	14 (20.9)	7 (43.8)	
Non-applicable (SBMA)	3 (3.7)	3 (4.5)	0 (0.0)	
CKD pre-allo-HCT	17 (20.7)	13 (19.4)	4 (25.0)	0.618
Hematological disease				
AL or MDS	52 (63.4)	41 (61.2)	12 (75.0)	0.910
LPS	18 (21.9)	15 (22.8)	3 (18.8)	
MPS	7 (8.6)	6 (9.0)	1 (6.2)	
SBMA or ID	3 (3.7)	3 (4.5)	0 (0.0)	
HL	2 (2.4)	2 (3.0)	0 (0.0)	
Pre-allo-HCT disease status				
Complete remission	48 (58.6)	40 (59.7)	8 (50.0)	0.779
Partial remission	22 (26.8)	17 (25.4)	5 (31.2)	
Disease progression	12 (14.6)	10 (14.9)	3 (18.8)	
Type of donor				
Unrelated donor	55 (67.0)	50 (74.6)	6 (37.5)	0.005
Identical family	13 (15.9)	7 (10.4)	6 (37.5)	
Haploidentical	13 (15.9)	10 (14.9)	3 (18.8)	
Umbilical cord	1 (1.2)	0 (0.0)	1 (6.2)	
Conditioning regimen				
Myeloablative (MAC)	27 (33.0)	22 (32.8)	5 (31.2)	0.903
Reduced intensity (RIC)	55 (67.0)	45 (67.2)	11 (68.8)	
Total body irradiation (TBI)	31 (37.8)	25 (37.3)	6 (37.5)	1.000
GVHD prophylaxis				
CI+ MMF	28 (34.1)	21 (31.3)	8 (50.0)	0.014
CI + MTX	5 (6.1)	2 (3.0)	3 (18.7)	
CI + CP	34 (41.5)	32 (47.8)	2 (12.6)	
CI + MMF + CP	15 (18.3)	12 (17.9)	3 (18.7)	
ATG use in GVHD prophylaxis	7 (8.5)	4 (6.0)	4 (25.0)	0.021
GVHD treated with prednisone ≥ 1 mg/kg	50 (61.0)	37 (55.2)	13 (81.3)	0.056
Serological risk of CMV				
Intermediate	34 (41.5)	32 (47.8)	3 (18.7)	0.035
High	48 (58.5)	35 (52.2)	13 (81.3)	
Days from allo-HCT to 1st CMV reactivation, median (IQR)	39.0 (31.3–49.0)	37.0 (30.5–48.0)	44.0 (42.5–57.0)	0.210

* One patient presented episodes in both cohorts (at-home program patients and inpatients). Abbreviations: AL (acute leukemia), allo-HCT (Allogeneic hematopoietic cell transplantation), ATG (Antithymocyte globulin), CI (Calcineurin inhibitor), CKD (Chronic kidney disease), CP (cyclophosphamide), GVHD (Graft-Versus-Host-Disease), HCT-IC (Hematopoietic Cell Transplantation Comorbidity Index Calculator), HL (Hodgkin lymphoma), ID (immunodeficiency), IQR (interquartile range), LPS (lymphoproliferative syndrome), MDS (myelodysplastic syndrome), MMF (mycophenolate mofetil), MPS (Myeloproliferative syndrome), MTX (methotrexate), SBMA (Severe bone marrow aplasia), SD (standard deviation).

**Table 2:** Foscarnet treatment data and clinical variables of the patients before and during foscarnet treatment.

Data, *n* (%)	Total Group (*n* = 82; 105 Episodes)	Home Care Unit Patients (*n* = 67; 88 Episodes)	Inpatients (*n* = 16; 17 Episodes)	*p* *
Number of CMV reactivations treated with FCN				0.163
1	67 (81.7)	53 (79.1)	15 (93.8)	
2	9 (11.0)	8 (11.9)	1 (6.2)	
3	5 (6.1)	5 (7.5)	0 (0.0)	
4	0 (0.0)	1 (1.5)	0 (0.0)	
5	1 (1.2) **	0 (0.0)	0 (0.0)	
CMV disease for each episode	12 (11.4)	10 (11.4)	2 (11.8)	0.962
CKD pre-FCN; *n* (%)	25 (30.5)	20 (29.9)	6 (37.5)	0.553
Neutrophil count (10^9^/L) pre-FCN; median (IQR)	1.7 (1.0–2.9)	1.7 (1.0–2.8)	2.4 (1.1–3.4)	0.692
CMV viral load (IU/mL) pre-FCN; median (IQR)	3363.5 (1647.5–8962.0)	3534.0 (1639.0–9228.0)	3082.0 (2436.0–7000.0)	0.638
Catheter bacteremia during treatment	10 (9.5)	9 (10.2)	1 (5.9)	0.576
Catheter thrombosis during treatment	3 (2.9)	2 (2.3)	1 (5.9)	0.414
Febrile syndrome without focus during treatment	14 (13.3)	11 (12.5)	3 (17.6)	0.568
Urinary tract infection, not BK virus related during treatment	8 (7.6)	5 (5.7)	3 (17.6)	0.090
BK virus cystitis during treatment	24 (22.9)	20 (22.7)	4 (23.5)	0.943
BK virus hemorrhagic cystitis during treatment	13 (12.4)	9 (10.2)	4 (23.5)	0.128
Respiratory tract infection during treatment	11 (10.5)	9 (10.2)	2 (11.8)	0.850
Sepsis during treatment	5 (4.8)	3 (3.4)	2 (11.8)	0.138
Occurrence or worsening of GVHD during treatment	21 (20.0)	16 (18.2)	5 (29.4)	0.289
Major cause to stop FCN				0.713
Toxicological consequences ***	23 (21.9)	20 (22.7)	3 (17.6)	
Response to drug	71 (67.6)	58 (65.9)	13 (76.5)	
Suspected viral resistance to FCN	6 (5.7)	5 (5.7)	1 (5.9)	
Other reasons	5 (4.8)	5 (5.7)	0 (0.0)	
Type of toxicological consequences *** that caused FCN discontinuation (23 episodes)				0.865
Genital ulcers or dysuria	9 (39.2)	8 (40.0)	1 (33.3)	
Impaired kidney function or proteinuria	11 (47.9)	9 (45.0)	2 (66.7)	
Genital ulcers + impaired kidney function, or proteinuria	2 (8.6)	2 (10.0)	0 (0.0)	
Digestive intolerance	1 (4.3)	1 (5.0)	0 (0.0)	
Nº of days of therapy; median (IQR)	15.0 (10.0–22.0)	15.0 (9.0–23.0)	14.0 (11.0–19.0)	0.692

* The statistics and frequencies (except for the item “number of CMV reactivations treated with FCN”) are based on episodes. ** One patient presented with four episodes in the at-home program and one in the inpatient cohort. *** Adverse effect exacerbated or initiated during treatment with foscarnet. It is not certain that this toxicity was produced by foscarnet or was produced only due to foscarnet. Abbreviations: CKD (Chronic kidney disease), CMV (cytomegalovirus), FCN (foscarnet), IQR (interquartile range).

**Table 3:** Toxicity per episode produced during treatment with foscarnet.

Data, *n* (%)	Total Group (*n* = 82; 105 Episodes)	Home Care Unit Patients (*n* = 67; 88 Episodes)	Inpatients (*n* = 16; 17 Episodes)	*p* *
Infusion reaction	17 (16.2)	13 (14.8)	4 (23.5)	0.370
Organ toxicity				
AKI (*n* de novo + *n* exacerbation of preexisting *CKD*); %	24 (17 + 7); 22.9	21 (16 + 5); 23.9	3 (2 + 1); 17.6	0.576
AKI de novo converts into CKD (considering 3 months post-FCN treatment’s serum creatinine)	8 (47.1)	7 (43.8)	1 (50.0)	0.768
Cardiac toxicity	0 (0.0)	0 (0.0)	0 (0.0)	-
Digestive toxicity	35 (33.3)	30 (34.1)	5 (29.4)	0.710
Dysuria without genito-urethral ulcer	11 (10.5)	11 (12.5)	0 (0.0)	0.123
Genito-urethral ulcers probably due to FCN	18 (17.1)	16 (18.2)	2 (11.8)	0.520
Liver toxicity	1 (1.0)	1 (1.1)	0 (0.0)	0.660
Neurological toxicity	1 (1.0)	1 (1.1)	0 (0.0)	0.660
Electrolyte imbalance				
Hypocalcemia (de novo or worsening)	33 (31.4)	23 (26.1)	10 (58.8)	0.008
Hypokalemia (de novo or worsening)	62 (59.0)	51 (58.0)	11 (64.7)	0.604
Hypomagnesemia (de novo or worsening)	42 (40.0)	37 (42.0)	5 (29.4)	0.330
Hyponatremia (de novo or worsening)	14 (13.3)	10 (11.4)	4 (23.5)	0.180

* The statistics and frequencies are based on episodes. Abbreviations: AKI (acute kidney disease), CKD (Chronic kidney disease), FCN (foscarnet).

**Table 4:** Economic analysis of foscarnet in the two cohorts.

Economic Cost (EUR); Median (IQR)	Home Care Unit Patients (*n* = 67; 88 Episodes)	Inpatients (*n* = 16; 17 Episodes)	*p* *
Cost for the entire episode with FCN for all patients	3254.1 (1805.8–6252.7)	8520.4 (5247.3–10,426.1)	0.003
Total cost of days with FCN (only HCU treatment days) or inpatients without major complications during hospitalization	2533.7 (1481.9–3954.6)	6324.9 (3184.0–7523.0)	<0.001
FCN cost/day (only HCU treatment days) or inpatients without major complications during hospitalization	290.0 (204.4–384.0)	477.0 (372.4–588.2)	<0.001

* The statistics are based on episodes. Abbreviations: FCN (foscarnet), IQR (interquartile range).

## Data Availability

Data are contained within the article and supplementary materials.

## Supplementary Materials

The following supporting information can be downloaded at: https://www.mdpi.com/article/10.3390/ph16121741/s1, Table S1: a description of the data collected for the study; Table S2: stages of acute kidney injury according to the KDIGO guideline; Table S3: summary of the dosing schedules for foscarnet indicated in the internal protocol; Table S4: classification of the toxicities collected in the study (except acute kidney injury) into the different degrees of severity according to the Common Terminology Criteria for Adverse Events (CTCAE) v5.0; Table S5: description and bibliography of the scales or index used in the manuscript.
