# Supplementary material for: At-Home Foscarnet Administration in Patients with Cytomegalovirus Infection Post-Allogeneic Stem Cell Transplantation: A Unicentric, Safe, and Feasible Program
Source: Pharmaceuticals (Basel). 2023 Dec 18;16(12):1741. doi: 10.3390/ph16121741 (PMC10748136; doi:10.3390/ph16121741)
Supplement: Supplementary file 1 [file pharmaceuticals-16-01741-s001.zip › pharmaceuticals-2751746-supplementary.pdf]

SUPPLEMENTARY MATERIAL

Table S1: description of the data collected for the study

Type of variable	Data collected
Demographic variable	Age, gender, weight, height
Allo-HCT variable	Pre-allo HCT disease status, type of allo-HCT, day of allo-HCT, conditioning regimen, GVHD prophylaxis, GVHD treated with prednisone ≥ 1mg/kg, post allo-HCT complications, disease re-evaluations after allo-HCT
Virologic variable	Serological risk of CMV, CMV viral load (IU/ml) pre- and post-treatment with FCN, CMV disease status (organ/system involved), days between Allo-HCT and 1st CMV reactivation, total episodes number of CMV reactivations or disease, treatment of previous CMV reactivations or disease
Clinical variable	Comorbidities, hematological disease, dialysis requirement, history of other serious infections, HCT-IC, Karnofsky Performance Status Scale, ECOG Performance Status Scale, Disease Risk Index.
Treatment data	Other therapy administered for CMV treatment pre-, during and post FCN therapy (if any), and reason of the switch/addition, patient location/s, other concomitant medications also received during treatment, doses and durations of FCN, reason for the end of treatment, type of catheter used, types of hydration and electrolyte replenishment serums.
Clinical complications pre, during and post treatment.	Infections, sepsis, febrile syndrome without focus, catheter thrombosis/infection, ICU admission or death, occurrence or worsening of GVHD. Emergency or hospital admission during treatment with FCN in at home program (reason, duration, return of the patient to HCU or not, completion of treatment with FCN during admission or not).
Analytical data	• Serum creatinine before allo-HCT, pre- and post-FCN, 3 months after FCN and worst value (the highest) during FCN treatment.<br>• Potassium, sodium, magnesium, phosphorus and albumin-adjusted calcium pre- and post-FCN, and worst value (the highest and the lowest) during FCN treatment.<br>• Transaminases, total bilirrubin, GGT and ALP pre- and post-FCN.<br>• Neutrophil, hemoglobin and platelet count pre- and post-FCN treatment.
Toxicity	• Renal toxicity: CKD, AKI, CKD exacerbation and/or proteinuria during FCN treatment.<br>• Hepatic toxicity: abnormal liver function or elevated transaminase during FCN treatment.<br>• Dyselectrolitemia: hipo- or hiperkalemia, -natremia, -magnesemia, phosphatemia or calcemia during FCN treatment.<br>• Gastrointestinal toxicity: Nausea, vomiting, heartburn, dysgeusia, ageusia, stomach pain, dysphagia, mouth ulceration and/or bloating during FCN therapy. Limited intake (less than 50% of usual) because of these symptoms.<br>• Urological toxicity: Dysuria, erythema and/or ulceration of the genitalia during FCN therapy. Presence or history of BK virus replication and associated symptomatology (cystitis, hemorrhagic or not).<br>• Cardiac toxicity: arrhytmias during FCN therapy.

Toxicity	• Neurological toxicity: Dizziness, headache or sleep disturbances during FCN therapy.<br>• Infusion reaction: nausea, skin reactions, respiratory symptoms, hypotension, paresthesia or chills or any other effects reported in clinical course during or after FCN administration.<br>Management performed (if any) and evolution/duration/resolution of the toxicity/reaction.<br>Mention in the clinical course that the toxicity/reaction could be produced by another drug. Presence of other toxic drugs (nephrotoxic, hepatotoxic, neurotoxic...) during treatment with FCN.<br>Patient's clinical condition (at the gastrointestinal level, at the renal level, at the hepatic level...) before and after the period of FCN treatment.<br>Toxicity/reaction severity level. Discontinuation of FCN treatment due to this toxicity/reaction.

Abbreviations: AKI (acute kidney injury), Allo-HCT (allogenic hematopoetic cell transplant), ALP (alkalin phosphatase), CKD (chronic kidney disease), CMV (citomegalovirus), FCN (foscarnet), GGT (gamma-glutamyltransferasa), GVHD (Graft-Versus-Host-Disease), HCT-IC (Hematopoietic Cell Transplantation Comorbidity Index Calculator), IU (international unit)

Table S2: stages of Acute Kidney Injury according to the KDIGO guideline

Stage	Definition
Stage 1 (AKI-1)	Increase in sCr 1.5-1.9 times baseline or ≥0.3 mg/dl increase within 48 hours.
Stage 2 (AKI-2)	Increase in sCr 2-2.9 times baseline.
Stage 3 (AKI-3)	Increase in sCr 3.0 times baseline, increase in serum creatinine to ≥4.0 mg/dl or initiation of renal replacement therapy.

Abbreviations: KDIGO (Kidney Disease Improving Global Outcomes), AKI (acute kidney injury), sCr (serum creatinine)

Table S3: summary of the dosing schedules for foscarnet indicated in the internal protocol

Foscarnet regimen for treatment of **CMV infection**	60 mg/kg/12h. The minimum duration will be 14 days, extending up to 21 days or more depending on whether the viral load at two weeks is undetectable, decreasing or unchanged.
Foscarnet regimen for treatment of **CMV disease**	60 mg/kg/8h or 90 mg/12h a minimum of 21 days, and whenever possible, 28 days (4 weeks).
Foscarnet **Maintenance** treatment	If maintenance treatment is chosen, it should be started after the end of induction treatment and would be recommended in the following cases:<br>a.- Patient who has presented ≥2 reactivations, separated in time by <4 weeks and who is undergoing corticosteroid treatment. It should be maintained up to 2 weeks after corticosteroid treatment (whenever possible).<br>b.- Patient who has presented CMV disease (either in the first reactivation or due to CMV or successive<br>infections, and especially if the patient is treated with corticosteroids).<br>c.- Maintenance treatment is debatable in patients who have had ≥ 2 CMV reactivations but separated by at least 4 weeks and who are not taking prednisone.<br>In case of using foscarnet as maintenance therapy, the recommended guideline will be: 60 mg/kg on Mondays, Wednesdays and Fridays. The chosen duration will be at the doctor's discretion.
Foscarnet regimen in case of **renal injury**	The adjustment will be the one recommended in the Summary of Product Characteristics of foscarnet of the European Medicines Agency.

Abbreviations: CMV (citomegalovirus)

Table S4: classification of the toxicities collected in the study (except acute kidney injury) into the different degrees of severity according the Common Terminology Criteria for Adverse Events (CTCAE) v5.0

Toxicity	Definition of the stages
Hepatic Toxicity	• **Alanine** or **aspartate aminotransferase increased**: grade I (>ULN* – 3.0 x ULN if baseline was normal; 1.5 – 3.0 x baseline if baseline was abnormal); grade II (>3.0 - 5.0 x ULN if baseline was normal; >3.0 - 5.0 x baseline if baseline was abnormal); grade III (>5.0 - 20.0 x ULN if baseline was normal; >5.0 - 20.0 x baseline if baseline was abnormal); grade IV (>20.0 x ULN if baseline was normal; >20.0 x baseline if baseline was abnormal)<br>• **Alkaline phosphatase** or **gamma glutamyl transferase increased**: grade I (>ULN* – 2.5 x ULN if baseline was normal; 2.0 – 2.5 x baseline if baseline was abnormal); grade II (>2.5 - 5.0 x ULN if baseline was normal; >3.0 - 5.0 x baseline if baseline was abnormal); grade III (>5.0 - 20.0 x ULN if baseline was normal; >5.0 - 20.0 x baseline if baseline was abnormal); grade IV (>20.0 x ULN if baseline was normal; >20.0 x baseline if baseline was abnormal)<br>• **Total bilirubin increased**: grade I (>ULN* - 1.5 x ULN if baseline was normal; > 1.0 - 1.5 x baseline if baseline was abnormal); grade II (>1.5 - 3.0 x ULN if baseline was normal; >1.5 - 3.0 x baseline if baseline was abnormal); grade III (>3.0 - 10.0 x ULN if baseline was normal; >3.0 - 10.0 x baseline if baseline was abnormal); grade IV (>10.0 x ULN if baseline was normal; >10.0 x baseline if baseline was abnormal)
Dyselectro-litemia	• **Hypocalcemia**: grade I (corrected serum calcium of <LLN**- 8.0 mg/dL; <LLN - 2.0 mmol/L; ionized calcium <LLN - 1.0 mmol/L; grade II (corrected serum calcium of <8.0 - 7.0 mg/dL; <2.0 - 1.75 mmol/L; ionized calcium <1.0 - 0.9 mmol/L; symptomatic); grade III (corrected serum calcium of <7.0 - 6.0 mg/dL; <1.75 - 1.5 mmol/L; ionized calcium <0.9 - 0.8 mmol/L; hospitalization indicated); grade IV (corrected serum calcium of <6.0 mg/dL; <1.5 mmol/L; Ionized calcium <0.8 mmol/L; life-threatening consequences)<br>• **Hypokalemia**: grade I (<LLN** - 3.0 mmol/L); grade II (Symptomatic with <LLN - 3.0 mmol/L; intervention indicated); grade III (<3.0 - 2.5 mmol/L; hospitalization indicated); grade IV (<2.5 mmol/L; life-threatening consequences)<br>• **Hypomagnesemia**: grade I (<LLN** - 1.2 mg/dL or <LLN - 0.5 mmol/L); grade II (<1.2 - 0.9 mg/dL or <0.5 - 0.4 mmol/L); grade III (<0.9 - 0.7 mg/dL; <0.4 - 0.3 mmol/L); grade IV (<0.7 mg/dL or <0.3 mmol/L; life-threatening consequences).<br>• **Hyponatremia**: grade I (<LLN** - 130 mmol/L); grade II (125-129 mmol/L and asymptomatic); grade III (125-129 mmol/L and symptomatic or 120-124 mmol/L regardless of symptoms); grade IV (<120 mmol/L; life-threatening consequences).
Renal toxicity	• **Proteinuria**: grade I (1+ proteinuria; urinary protein ≥ULN - <1.0 g/24 h); grade II (2+ and 3+ proteinuria; urinary protein 1.0 - <3.5 g/24 h); grade III (urinary protein >=3.5 g/24 h; 4+ proteinuria); grade IV (-)
	• **Nausea:** grade I (loss of appetite without alteration in eating habits); grade II (oral intake decreased without significant weight loss, dehydration or malnutrition); grade III (inadequate oral caloric or fluid intake; tube feeding, TPN, or hospitalization indicated); grade IV (-).<br>• **Vomiting:** grade I (intervention not indicated); grade II (outpatient IV hydration; medical intervention indicated); grade III (tube feeding, parenteral

	nutrition, or hospitalization indicated); grade IV (life-threatening consequences).
GI toxicity	• **Gastrointestinal pain:** grade I (mild pain); grade II (moderate pain; limiting instrumental ADL); grade III (severe pain; limiting self-care ADL); grade IV (-)<br>• **Dysgeusia or ageusia:** grade I (altered taste but no change in diet); grade II (altered taste with change in diet; noxious or unpleasant taste; loss of taste); grade III-IV (-).<br>• **Others GI disorders:** grade I (asymptomatic or mild symptoms; clinical or diagnostic observations only; intervention not indicated); grade II (moderate; minimal, local or noninvasive intervention indicated; limiting age-appropriate instrumental ADL); grade III (severe or medically significant but not immediately life-threatening; hospitalization or prolongation of existing hospitalization indicated; limiting self-care ADL); grade IV (life-threatening consequences; urgent intervention indicated).
Urological toxicity	• **Dysuria**: grade I (present); grade II-IV (-)<br>• **Urinary tract pain:** grade I (mild pain); grade II (moderate pain; limiting instrumental ADL); grade III (severe pain; limiting self-care ADL); grade IV (-).<br>• **Skin ulceration:** grade I (combined area of ulcers <1 cm; nonblanchable erythema of intact skin with associated warmth or edema); grade II (combined area of ulcers 1-2 cm; partial thickness skin loss involving skin or subcutaneous fat); grade III (combined area of ulcers >2 cm; full-thickness skin loss involving damage to or necrosis of subcutaneous tissue that may extend down to fascia); grade IV (any size ulcer with extensive destruction, tissue necrosis, or damage to muscle, bone, or supporting structures with or without full thickness skin loss).<br>• **Other:** grade I (asymptomatic or mild symptoms; clinical or diagnostic observations only; intervention not indicated); grade II (moderate; minimal, local or noninvasive intervention indicated; limiting age-appropriate instrumental ADL); grade III (severe or medically significant but not immediately life-threatening; hospitalization or prolongation of existing hospitalization indicated; limiting self-care ADL); grade IV (life-threatening consequences; urgent intervention indicated)
Cardiac toxicity	• **Arrhythmia:** grade I (Asymptomatic, intervention not indicated); grade II (non-urgent medical intervention indicated); grade III (urgent intervention indicated); grade IV (life-threatening consequences; hemodynamic compromise)
Neurological toxicity	• **Dizziness:** grade I (mild unsteadiness or sensation of movement); grade II (moderate unsteadiness or sensation of movement; limiting instrumental ADL); grade III (severe unsteadiness or sensation of movement; limiting self-care ADL); grade IV (-)<br>• **Headache:** grade I (mild pain); grade II (moderate pain; limiting instrumental ADL); grade III (severe pain; limiting self-care ADL); grade IV (-)<br>• **Insomnia:** grade I (mild difficulty falling asleep, staying asleep or waking up early); grade II (moderate difficulty falling asleep, staying asleep or waking up early); grade III (severe difficulty in falling asleep, staying asleep or waking up early)<br>• **Somnolence:** grade I (mild but more than usual drowsiness or sleepiness); grade II (moderate sedation; limiting instrumental ADL); grade III

	(obtundation or stupor); grade IV (life-threatening consequences; urgent intervention indicated).
Infusion reaction	• **General disorder** or **chills:** grade I (asymptomatic or mild symptoms; clinical or diagnostic observations only; intervention not indicated); grade II (moderate; minimal, local or noninvasive intervention indicated; limiting age-appropriate instrumental ADL); grade III (severe or medically significant but not immediately life-threatening; hospitalization or prolongation of existing hospitalization indicated; limiting self-care ADL); grade IV (life-threatening consequences; urgent intervention indicated)<br>• **Flu like symptoms:** grade I **(**mild flu-like symptoms present); grade II (moderate symptoms; limiting instrumental ADL); grade III (severe symptoms; limiting self-care ADL); grade IV (-)<br>• **Paresthesia**: grade I (mild symptoms); grade II (moderate symptoms; limiting instrumental ADL); grade III (severe symptoms; limiting self-care ADL); grade IV (-)<br>• **Rash acneiform:** grade I (papules and/or pustules covering <10% BSA, which may or may not be associated with symptoms of pruritus or tenderness); grade II (papules and/or pustules covering 10 - 30% BSA, which may or may not be associated with symptoms of pruritus or tenderness; associated with psychosocial impact; limiting instrumental ADL; papules and/or pustules covering > 30% BSA with or without mild symptoms); grade III (papules and/or pustules covering >30% BSA with moderate or severe symptoms; limiting self-care ADL; associated with local superinfection with oral antibiotics indicated); grade IV (life-threatening consequences; papules and/or pustules covering any % BSA, which may or may not be associated with symptoms of pruritus or tenderness and are associated with extensive superinfection with IV antibiotics indicated)

Abbreviations: ADL (activities of daily living); BSA (body surface area); GI (gastrointestinal); LLN (lower limit or normal); ULN (upper limit of normal)

*The ULN used were 40.0 U/L for alanine, aspartate aminotransferase or gamma-glutamyl transferase, 116.0 U/L for alkaline phosphatase and 1.2 mg/dL for total bilirubin.

**The LLN used were 135.0 mmol/L for sodium, 3.5 mmol/L for potassium, 1.8 mg/dL for magnesium and 8.5 mg/dL for serum calcium.

Table S5: description and bibliography of the scales or index used in the manuscript.

Scale or index	Description and bibliography
HCT-IC	It is a comorbidity index that provides information with regard to the overall as well as NRM risk at 2-years a patient is likely to experience after hematopoietic cell transplantation. The index classifies patients into three risk groups according to the scored obtained: low risk or score of 0 (NRM 14%, OS 71%), intermediate risk or score of 1-2 (NRM 21%, OS 60%) and high risk or score of ≥3 (NRM 41%, OS 34%).<br>- Sorror, M.L., Maris, M.B., Storb, R., Baron, F., Sandmaier, B.M., Maloney, D.G., Storer, B. Hematopoietic cell transplantation (HCT)-specific comorbidity index: a new tool for risk assessment before allogeneic HCT. *Blood*. **2005**; 106: 2912-2919. doi:10.1182/blood-2005-05-2004
Karnofsky Performance Status Scale	Scale used for performance assessment of cancer patients. The scale is ranging from full wellbeing (100%) to death (0%), decreasing ten points at each level. Patients are divided into three groups; Group A (100%–80%) can independently perform daily activities, Group B (80%– 60%) can perform daily activities with little help or effort, Group C (<60%, requires considerable or continuous assistance).<br>- Peus, D., Newcomb, N., Hofer, S. Appraisal of the Karnofsky performance status and proposal of a simple algorithmic system for its evaluation. *BMC Med Inform Decis Mak*. **2013**; 13: 1-7. doi: 10.1186/1472-6947-13-72.
ECOG Performance Status Scale	Scale used for performance assessment of cancer patients. It essentially uses 2 elements for patient categorization: ability to ambulate and need for care. The scale is ranging from normal functioning (ECOG = 0) to death (ECOG = 5). With a score of 2 the patient is capable of self-care as well as walking more than 50% of waking hours but unable to perform any type of work.<br>- Sørensen, J., Klee, M., Palshof, T., Hansen, H.H. Performance status assessment in cancer patients. An inter-observer variability study. *Br J Cancer*. **1993**; 67: 773–775. doi: 10.1038/bjc.1993.140.
Disease Risk Index	It is a validated tool to categorize groups of patients undergoing allo-HCT for hematologic malignancy by disease risk, predicting 2-year OS probabilities. The index classifies patients into different categories of risk: low (66% OS), intermediate (51% OS), high (33% OS) and very high (23% OS).<br>- Armand, P., Kim, H.T., Logan, B.R., Wang, Z., Alyea, E.P., Kalaycio, M.E., Maziarz, R.T., Antin, J.H., Soiffer, R.J., Weisdorf, D.J., Rizzo, J.D., Horowitz, M.M., Saber, W. Validation and refinement of the Disease Risk Index for allogeneic stem cell transplantation. *Blood*. **2014**; 123: 3664-3671. doi: 10.1182/blood-2014-01-552984.

Abbreviations: Allo-HCT (allogenic hematopoetic cell transplant), HCT-IC (Hematopoietic Cell Transplantation Comorbidity Index Calculator), NRM (non-relapse mortality), OS (overall survival).
